# Journey of micronanoplastics with blood components

**DOI:** 10.1039/d3ra05620a

**Published:** 2023-10-27

**Authors:** Durgalakshmi Rajendran, Natarajan Chandrasekaran

**Affiliations:** a Centre for Nanobiotechnology, Vellore Institute of Technology Vellore 632014 Tamil Nadu India nchandrasekaran@vit.ac.in nchandra40@hotmail.com +91 416 2243092 +91 416 2202624

## Abstract

The entry of micro- and nanoplastics (MNPs) into the human body is inevitable. They enter blood circulation through ingestion, inhalation, and dermal contact by crossing the gut–lung–skin barrier (the epithelium of the digestive tract, the respiratory tract, and the cutaneous layer). There are many reports on their toxicities to organs and tissues. This paper presents the first thorough assessment of MNP-driven bloodstream toxicity and the mechanism of toxicity from the viewpoint of both MNP and environmental co-pollutant complexes. Toxic impacts include plasma protein denaturation, hemolysis, reduced immunity, thrombosis, blood coagulation, and vascular endothelial damage, among others, which can lead to life-threatening diseases. Protein corona formation, oxidative stress, cytokine alterations, inflammation, and cyto- and genotoxicity are the key mechanisms involved in toxicity. MNPs change the secondary structure of plasma proteins, thereby preventing their transport functions (for nutrients, drugs, oxygen, *etc.*). MNPs inhibit erythropoiesis by influencing hematopoietic stem cell proliferation and differentiation. They cause red blood cell and platelet aggregation, as well as increased adherence to endothelial cells, which can lead to thrombosis and cardiovascular disease. White blood cells and immune cells phagocytose MNPs, provoking inflammation. However, research gaps still exist, including gaps regarding the combined toxicity of MNPs and co-pollutants, toxicological studies in human models, advanced methodologies for toxicity analysis, bioaccumulation studies, inflammation and immunological responses, dose–response relationships of MNPs, and the effect of different physiochemical characteristics of MNPs. Furthermore, most studies have analyzed toxicity using prepared MNPs; hence, studies must be undertaken using true-to-life MNPs to determine the real-world scenario. Additionally, nanoplastics may further degrade into monomers, whose toxic effects have not yet been explored. The research gaps highlighted in this review will inspire future studies on the toxicity of MNPs in the vascular/circulatory systems utilizing *in vivo* models to enable more reliable health risk assessment.

## Abstract 2

Micronanoplastics (MNPs) interact with blood components, resulting in anemia, cardiovascular diseases, *etc.* Research gaps include toxic impacts of real-world MNPs, monomers, co-pollutants complex, and so on, emphasizing the need for more research.

## Graphical abstract

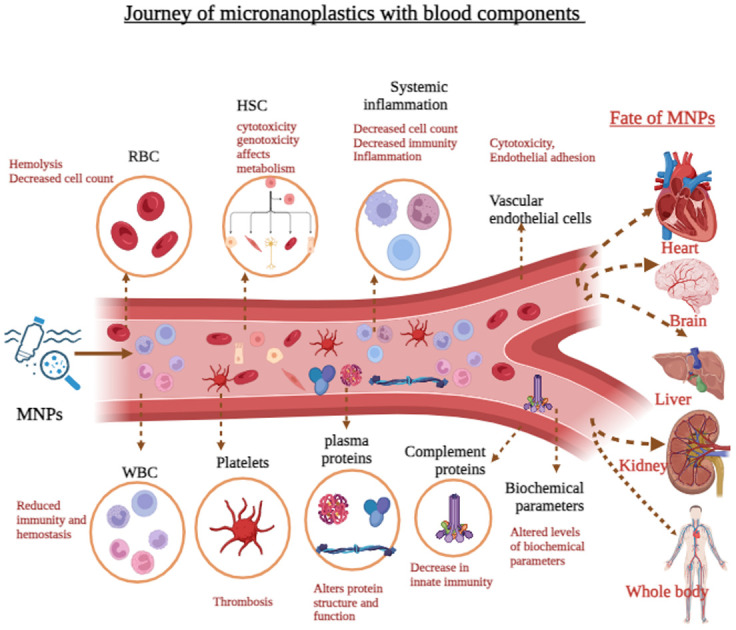

## 1. Introduction

Plastic is a ubiquitous material in our daily lives that has become unavoidable. Due to its exceptional physical and chemical properties—including flexibility, infrangibility, low density, low electrical conductivity, *etc*.—as well as its low cost, plastic has essentially supplanted the usage of wood and metal in many applications.^1^ The most common types of plastics used globally in medical, industrial, and consumer products are polypropylene (PP; medical and electronic equipment, straws, furniture),^2^ polyethylene (PE), which is mainly used in its low-density form (LDPE; bin bags, plastic wrap, shopping bags) and high-density form (HDPE; irrigation and drainage pipes, shampoo bottles, detergent bottles),^3^ polyvinyl chloride (PVC; electrical cable insulation, doorframes, toys, pipes),^4^ polystyrene (PS; foam food containers, plastic containers, rigid trays, audio and video cassettes, lids, and tumblers)^5^ and polyethylene terephthalate (PET; bottles, vehicle tires, conveyers, drive or seat belts, food trays).^6,7^ Plastic is beneficial; however, it takes hundreds of years to decompose and is not biodegradable. The natural environment is gradually becoming contaminated with plastic debris, including used plastic bags, bottles, and containers; this contamination has emerged as a critical global issue causing environmental stress and uncontrollable harm to living systems.^8^

A prognosis for 2050 predicts that the amount of plastic garbage in landfills and/or the environment will reach close to 12 000 Mt globally, and in seas and oceans, it will surpass fish.^9^ Plastic fragments degrade when exposed to ultraviolet light, weathering, and biodegradation, resulting in the formation of a heterogeneous mixture of microplastics (MPs) and nanoplastics (NPs). These plastic residues are classified as large microplastics (5 mm to 1 mm), small microplastics (1 mm to 1 μm), and nanoplastics (<1 μm).^10^ The ocean and other bodies of water are significantly contaminated by these microplastics. Thompson *et al.* formally coined the term “microplastic” (MP) in 2004, in response to the growing problem of plastic pollution in the seas, and stated that these lightweight fragments follow atmospheric currents and are dispersed globally.^11^ Microplastics are therefore present in every environmental compartment (air, soil, and bodies of water) and their presence is increasing at an alarming rate. MPs can be classified into two groups: primary and secondary. Primary MPs are directly released into the environment as microscale plastics (<5000 μm), such as in cosmetic products, toothpaste, pharmaceutical vectors (nanovehicles), *etc.*, The degradation of these primary MPs by mechanical or natural processes, *i.e.*, weathering, UV radiation, or other atmospheric currents, leads to the synthesis of secondary MPs^12^ Due to their smaller size, high mechanical strength, huge surface area, and high chemical activity, MPs and NPs have different physical and chemical properties compared to the bulk materials. Plastics often have a surface charge as a result of the functionalization of plastic products during production to obtain the desired material properties. Common surface modifications include amino groups (NH_2_) to give positively charged surfaces, carboxyl groups (COOH) to give negatively charged surfaces, and non-functionalized raw plastic surfaces without any surface modification at all.^13,14^ A single plastic particle may have multiple distinct surface chemistries as a result of the environmental degradation process itself. Additionally, MNPs act as vectors for other environmental co-pollutants such as pesticides, heavy metals, pharmaceuticals, organic pollutants, *etc.*, by adsorbing them onto their surface *via* physical and chemical interactions.^15–17^ This will affect their environmental fate and behavior, as well as their interactions with biological systems.^18^ MNPs, whether manufactured or resulting from environmental degradation or leaching from medical devices and utilities, can enter living beings through ingestion, inhalation, or dermal pathways. Tiny organisms such as zooplankton and aquatic species such as fish, whales, turtles, *etc.*, can ingest MNPs and co-pollutants, leading to a decrease in their population and the collapse of entire ecosystems with further trophic transfer to humans.^19–22^ The ingestion of these particles has thus raised concerns about their potential impact on human health.

MNPs and pollutant complexes are ubiquitous and can enter the human body *via* inhalation,^23–25^ ingestion,^26–28^ or skin contact.^29^ Due to their smaller size, they are able to cross the gut–lung–skin barrier,^30–32^ which refers to the epithelial lining of the intestine, lung, and cutaneous layer.^33^ By crossing this barrier, they enter the bloodstream and are distributed to other organs. Several investigations have indicated that MNPs are hazardous to humans at all levels, from the organs (heart,^34^ liver,^35–37^ lungs,^31,32,38^ brain,^39,40^ placenta,^41–44^*etc.*) to the genome (DNA).^45–48^ However, the distributor of these particles to other organs is the blood and blood components.^41,49,50^ Hence, it is essential to analyze their interactions with blood components, as blood components are essential for many physiological activities and play an important part in preserving human health. They have major roles in the transportation of oxygen, nutrients, hormones, waste, external agents, *etc.* (plasma proteins and red blood cells (RBCs)),^51,52^ defense (white blood cells, WBCs),^53^ hemostasis (platelets),^54,55^ control of temperature and pH, and fluid management^52^ in the body. Their harmful impacts on blood components can lead to several abnormalities, such as hemolytic diseases,^51^ clotting and cardiovascular diseases,^56^ and downregulated immune response.^53^ Research gaps exist regarding the fate and detrimental impact of MNPs in the bloodstream due to a lack of epidemiological statistics and practical toxicological approaches, as well as technological limitations for assessing MNPs. Despite these limitations, available *in vitro* and *in vivo* investigations have suggested that MNPs could be harmful to blood components.^21,48,57–60^ This in-depth review article surveys the existing literature on the representative toxic impacts of MNPs on blood components, which will act as an eye-opener to other researchers to enable the filling of research gaps regarding toxicological data for MNPs in the vascular/circulatory system.

## 2. Methodology

The purpose of this review is to collect literature with a high-level scope to identify relevant studies on the effect of nanoplastics and microplastics on blood and blood components and consolidate it into a comprehensive overview of the existing literature in this area. The PubMed, Google Scholar, Web of Science, ScienceDirect, and Scopus databases were searched for potential papers using search terms such as microplastic, nanoplastic, co-pollutants, environmental effects, human exposure, blood, blood components, and organ toxicity. Peer-reviewed journal articles, books, reports, conference abstracts, and papers from the whole database record were included in this critical evaluation; consequently, no time frame was specified. Additionally, publications in languages other than English, unpublished literature, and sources from predatory or suspicious journals were not taken into account for this review. To prepare this article, data from the search results were processed, divided into categories, and presented under relevant headings. The software program Mendeley was used to cite the references.

## 3. Plastic pollution

MNPs are massive environmental pollutants to which humans and other environmental organisms are exposed over an extended period of time—almost their entire existence. Single-use plastic bags are the main source of plastic pollution, since they ultimately wind up in landfills, oceans, and other waterways, endangering our ecosystem.^61^ Plastic pellets, cosmetics with microbeads, paint, baby toys, food and beverage containers, sewage sludge, and tires are some of the primary sources of environmental MNPs. Municipal wastes, such as plastic bags, water bottles, fishing nets, and agricultural film are secondary sources of MNPs.^62^ These plastics are degraded in the environment by means of biodegradation (mediated by environmental microorganisms) and non-biodegradation processes (thermal (high temperature), physical (weathering), photodegradation (UV from sunlight), and hydrolysis), eventually becoming MPs and NPs.^63^ Additionally, MPs and NPs are employed in bioimaging,^64^ personal care products,^65^ and medicine delivery.^66^ The lack of handling limitations results in higher occupational exposure during the production of primary MNPs, and improper disposal of their waste pollutes the environment.^13^ Depending on the degree of degradation, MNPs follow biogeochemical cycles involving the atmosphere, lithosphere (terrestrial), and hydrosphere (aquatic system), all of which are interconnected.^67^

Terrestrial habitats are regarded as enormous MNP reservoirs, as MNPs have been reported to be present in agricultural soil samples from Germany (0.34 particles per kg of soil),^68^ Shanghai (256.7 ± 62.2 particles per kg),^69^ China (320–12 560 particles per kg of soil)^70^ and in many other locations worldwide.^71–73^ Sewage sludge utilized as fertilizer is the main source of plastic pollution in agricultural lands, as evidenced by sludge analysis in China (5553–13 460 particles of MNPs per kg of sludge).^74^ MNPs from the terrestrial environment reach the aquatic environment by natural erosion, surface runoff,^75^ or escape from wastewater treatment plants.^76^ MNP debris has been found in abundance in aquatic environments in the United States (30.8 ± 12.1 particles per L),^77^ Siberian Seas (0–0.045 particles per m^2^),^78^ India (2–178 particles per m^2^), North Atlantic subtropical gyre (13–501 particles per m^2^),^79,80^ China (20–340 particles per kg),^81^ Sweden (0.18–0.92 particles per m^2^),^82^ and Canada (0.19 particles per m^2^).^83^ In terms of atmospheric air currents, studies have reported the presence of airborne microplastics in Indonesia (132.75–174.97 particles per m^3^),^84^ China (4.18 particles per m^3^),^85^ Iran (0.3–1.1 particles per m^3^)^86^ and many other countries. These widespread MNPs acquire different physiochemical characteristics, such as heterogenous size, shape, surface roughness, hydrophobicity, and surface functionalization, through geochemical cycles and weathering, as well as by absorbing co-existing contaminants.^87^ These complex MNPs are then ingested by smaller organisms and make their entry into the food chain.^28,88^

## 4. Co-existence of contaminants

MNPs have evolved into excellent vehicles for the transport of heavy metals,^89^ persistent organic pollutants (POPs),^90^ microbial pathogens,^91^ and metabolized or unmetabolized pharmaceuticals^92–94^ from an aquatic environment to other ecosystems. These environmental contaminants are produced through consumerism and commercialism.^95^ Heavy metals and hydrocarbon chemicals are released into the environment from mining and mineral processing.^16,96,97^ Farming methods are the root cause of the environmental presence of harmful pesticides, nitrates, phosphates, and mineral salts.^22,98^ Recent studies of wastewater treatment plant (WWTP) effluent and surface water have found extremely high quantities of pharmaceutical pollutants.^99,100^ However, all of these organic and inorganic compounds, including pesticides, heavy metals, hydrocarbons, minerals, and pharmaceuticals, are discharged into the environment from manufacturing industries through wastewater effluents. Domestic usage of all the above-mentioned chemicals is another major cause of their presence in garbage and sewage.^95^ MNPs are more likely to act as vectors of concurrent pollutants and pose a major threat to the environment due to their small size, high surface-to-volume ratio, and high reactivity. Other important physiochemical properties, such as their non-polar nature (polyethylene (PE), polypropylene (PP), polystyrene (PS)^101^), amorphous structure (polyvinyl chloride (PVC), PS), and glass transition temperature of ∼90 °C (PVC, PS),^17^ enable them to adsorb co-pollutants by forming chemical interfaces, such as van der Waals bonds, hydrophobic interactions, or intraparticle diffusion.^17^ As a result, unanticipated environmental risks may appear due to the “Trojan Horse” effect. For example, PE and PS adsorb sulfamethoxazole,^102^ PP and PS adsorb tris-(2,3-dibromopropyl) isocyanurate and hexabromocyclododecanes,^103^ and PP, PE, PS, and PVC adsorb tylosin.^104^ PVC^105^ and PE^106^ have the capacity to adsorb the hydrophilic drug ciprofloxacin. Among the plastics, polystyrene nanoplastics (PSNPs) adsorb a wide range of environmental contaminants such as pharmaceuticals (oxytetracycline,^107^ ciprofloxacin^105^), POPs (polychlorinated biphenyls (PCBs), polycyclic aromatic hydrocarbons (PAHs), and dichlorodiphenyltrichloroethane (DDT)^108^), pesticides (fipronil,^109^ triadimenol (TRI), myclobutanil (MYC) and hexaconazole (HEX)^98^), heavy metals (arsenic^110^) and personal care products.^111^ PS adsorbs the heavy metal arsenic through hydrogen bonds, hydroxyl production, and surface adhesion, and transports it to the next habitat.^110^ Several kinetic studies on the interactions between microplastics and medicinal products have been primarily concerned with environmental issues. As a result of isotherm prediction, many successful attempts to model pollutant adsorption on MNPs have been reported.^112–115^

## 5. Potential impact of MNPs and co-pollutants on ecosystems

The negative impacts of accumulated MNPs on soil systems are unpredictable. Through a variety of toxic mechanisms, including bioaccumulation, oxidative stress, inflammation, metabolic disorders, gut microbiota dysbiosis, genotoxicity, neurotoxicity, and reproductive toxicity, MNPs significantly harm soil fauna, mostly earthworms and nematodes.^116^ This will have a negative effect on ecological processes such as nutrient cycling, energy flow, and litter decomposition, and poses numerous risks to the environment.^117^ Additionally, MNPs interact with other pollutants such as pesticides and other organic pollutants throughout their existence, increasing their potential for harm and severely damaging the terrestrial biota.^118^ The physiochemical characteristics of soil are affected by these complexes, leading to groundwater contamination, which inhibits plant growth and lowers overall productivity.^119^ Additionally, it has been noted that MNPs suppress the proliferation of microorganisms, including certain yeast, bacteria, and algae, which affects their crucial roles in various habitats.^120^ Numerous studies have been conducted on the health effects of MNPs on freshwater and marine organisms. In marine benthic animals (bivalves), such as mussels and oysters, MNPs have been observed to obstruct the digestive tract, which frequently results in decreased appetite, malnutrition, and fatalities.^121,122^ Bivalves are filter feeders^123^ and mistakenly consume plastic particles as food; these particles are absorbed into their circulatory system and distributed throughout the body, affecting growth and reproduction.^124^ Numerous studies on various marine creatures, including lugworms^125^ and oyster larvae,^126^ have revealed variable effects of plastic particles on growth and reproduction. In freshwater settings, studies have examined how MNPs affect zebrafish and *Daphnia magna*. MNPs can be consumed by *Daphnia magna* and alter their feeding and excretion rate.^127^ MNPs have been found to accumulate in the tissues of zebrafish and cause organ toxicity, inflammation, lipid accumulation, oxidative stress, and metabolic effects.^128^ Another study reported that 21 day exposure to 10 000 particles per L of realistic MNPs increased oviposition and secondary patellar aneurysms in Japanese medaka.^129^

In addition to the direct toxicity of nanoplastics, it is important to take into account the possible toxicity of MNPs and coronated pollutants. Several combined toxicity studies of nanoplastics and co-pollutants have been conducted using aquatic organisms,^130–134^ mouse models,^37,135^ and cell lines.^136,137^ Oxidative stress brought on by exposure to pollutants can result in the generation of reactive oxygen species (ROS) and impairment of the antioxidant defense mechanisms in organisms.^138^ Recent studies have also revealed that when *Scenedesmus obliquus* and zebrafish (*Danio rerio*) were exposed to both PSNPs and naturally occurring acidic organic polymers (NAOP), such as fulvic acid and humic acid, growth inhibition of the algae and oxidative stress in the zebrafish were observed.^139^ Ibuprofen showed synergistic toxicity with PSNPs through suppressing growth in *Chlorella pyrenoidosa*, indicating pharmaceutical-assisted toxicity.^134^ Antibiotics—doxycycline, in particular—have been shown to make microplastics more harmful to *Tetraselmis chuii* at concentrations as low as a few parts per million (ppm).^133^ Studies found that PS complexed with roxithromycin exacerbated the effects of oxidative stress in red tilapia (*Oreochromis niloticus*) and *Daphnia magna*.^132^ Similarly, in *Misgurnus anguillicaudatus*, venlafaxine complexed with PVC-MPs boosted superoxide dismutase activity.^140^ Sulfamethazine, a different medication, also had a detrimental effect on the marine medaka (*Oryzias melastigma*) antioxidant system when it co-occurred with PSNPs.^130^ In a similar way, amplified lipid peroxidase activity was observed due to exposure to MNPs coronated with sertraline in *Tegillarca granosa*,^131^ florfenicol in *Corbicula fluminea*,^141^ and cefalexin in *Pomatoschistus microps*.^142^ After entering and accumulating in these aquatic organisms, MNPs and co-pollutants enter the food web, as they are consumed by humans.^143^

## 6. Potential impact of MNPs and co-pollutants on human health

**Fig. 1:**
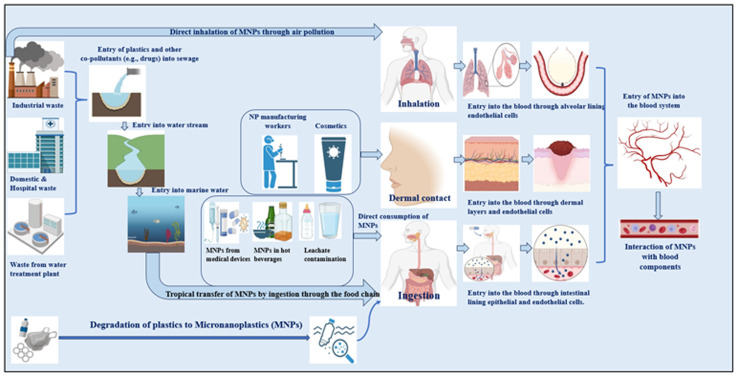
Routes of entry of micronanoplastics into the human body.

MNPs are predicted to enter the body mostly through ingestion, inhalation, and skin contact^144^ (Table 1 and Fig. 1). Concern over human exposure to these particles has been raised by recent discoveries of MPs in seafood,^28^ entering through the food chain, and in drinking water (with food and air as additional sources of exposure).^9^ The inside lining of single-use paper cups was discovered to leach MNPs into hot water and hot beverages. In general, HDPE and co-polymers are used to generate these hydrophobic plastic liners, which constitute 5–10% of the total weight. Hot water exposure releases microplastics, toxic heavy metals such as lead, chromium, and cadmium, and ions including fluoride, chloride, sulfate, and nitrate,^145^ resulting in an average CDI (chronic daily intake) by humans of 0.03 ± 0.025 mg of microplastic per kilogram of body weight per day and 7.04 ± 8.8 g fluoride per kilogram of body weight per day.^145,146^

**Table 1:** Routes of entry of micronanoplastics into the human body

Route of entry	Source of MNP contamination	Ref.
Ingestion	Drinking water	6, 26, 147 and 148
	Seafood – fish	28 and 149
	Salt	24 and 150
	Tea bags	151
	Honey	27
	Hot beverages from paper cups	146
	Foods stored in plastic containers	152
Inhalation	Polluted air/dust	24, 153, 23, 38 and 154
Dermal contact	Cosmetics	29
	Workers in nanoparticle manufacturing industries	25

To forecast the potential detrimental effects of MNPs on human health, researchers are continuing to use mammalian animal models. In the same vein, various *in vivo* and *ex vivo* investigations have shown the toxicity of MNPs in living cells.^155^ The cytotoxic effect of MNPs has been confirmed on numerous cell lines, for example, lung cells,^32^ intestinal cells,^156,157^ and many others. Cationic polystyrene nanoplastics (60 nm) were proven to affect cell viability and enhance inflammatory gene expression in gastric cell lines,^158^ macrophage^159^ cell lines, and epithelial cells.^160^ The bioavailability of polystyrene MNPs in rat mammalian models was shown to be about 4% in the blood, bone marrow, liver, and spleen.^161^ Mice treated with PSMPs for 28 days exhibited the presence of PSMPs in the liver, kidney, and stomach.^162^ In a different study, mice exposed to PS showed significant changes in the diversity and abundance of intestinal biota, as well as a decrease in intestinal mucus.^163,164^ After receiving 1 to 10 mg of PS NPs per kg of body weight per day for five weeks, adult male Wistar rats displayed decreased locomotor activity.^165^

In addition to the toxicity of pristine MNPs to organisms, studies have also proven the toxic impacts of complexes of MNPs coronated with co-pollutants; however, only a few such studies exist. According to a recent study, the entry of environmental pollutants such as polychlorinated biphenyls (PCBs) into the body can disrupt and change the natural balance of a mother's milk, which can have negative health effects on nursing infants, including allergies and endocrine disorders, as well as impaired neurodevelopment.^166^ In previous investigations, it was discovered that fish experience endocrine disruption when the emerging pollutant metformin hydrochloride is released widely into wastewater treatment facilities.^167^ Functionalized PSNPs (pristine, carboxy, and amine), when combined with arsenic and methylmercury, altered transcription of oxidative stress genes and induced apoptosis in a brain-derived cell line (SaB-1) of seabream fish (*Sparus aurata*).^136^ The heavy metal arsenic, when complexed with PSNPs, induced apoptosis, pyroptosis, and excessive autophagy in the mouse liver.^37^ Compared to those exposed to pristine particles, mice exposed to MNPs and organophosphorus flame retardants simultaneously experienced higher levels of oxidative stress, neurotoxicity, and metabolic disturbance.^168^ Synthetic pollutants such as phthalates and bisphenols are found in many plastic products, including food containers, personal care items, and healthcare items. For example, phthalates, which may make up to 50% of the total mass of plastics, are additives that are frequently found in nanoplastics.^169^ They have the ability to modify the endocrine system and are hence termed endocrine-disrupting compounds, which can subsequently lead to the development of breast cancer.^170^ These additives frequently include known carcinogens such as dioxins, polycyclic aromatic hydrocarbons, halogenated flame retardants, and heavy metals, and may be released from the polymers.^171^ The inherent properties of MNPs, the leaching of additives, and the release of persistent sorbed pollutants combine to cause the adverse effects of MNPs on the environment and living beings.^61^ In accordance with the outcomes of these experiments, it is believable to anticipate that MNPs and co-pollutants may have an impact on human health.^172^

## 7. Interaction with the circulatory system

The toxicological effects of MNPs have been well investigated, but research on how these particles affect blood components leaves much to be explored. MNPs (particularly <1 μm MNPs) may pass through barrier cells such as bronchial epithelial cells and endothelial cells and enter the circulatory system.^173^ They have demonstrated their ability to translocate through the intestinal barrier and impact lymphocyte cells in *in vitro* models of the intestinal barriers.^30^ A hematological investigation on mice demonstrated that PSNPs with a size of 5 μm are transported by the bloodstream to the stomach, intestines, liver, and other digestive organs, as well as the bones of the animals.^174^ In mammalian animal models, it has been demonstrated that plastic particles 100 nm and smaller translocate *via* the pulmonary and gastrointestinal routes of exposure into tissues and the circulatory system.^175^ This leads to a presumption that after internalization, the MNPs can interact with blood cell components and the vascular endothelium, which might lead to reactions such as MNP–blood interactions, cell damage, acute inflammation, and chronic inflammation.^56^ Therefore, hematopoietic cells, red cells, white cells, platelets, complement proteins, and plasma proteins have been hypothesized to be targets of MNP exposure.

However, if MNPs enter *via* an intravenous channel, the constituent parts of blood will come into contact with them first. This interaction may result in a number of linked pathophysiological events, such as oxidative stress, genotoxicity, and cytotoxicity.^176^ The term “cytotoxicity” describes the capacity of a substance to harm or destroy cells. Genotoxicity is the term used to describe the capacity of a substance to harm DNA, resulting in mutations and other genetic alterations.^177^ Researchers have revealed that exposure to polystyrene microplastics significantly harmed human white blood cells, peripheral blood mononuclear cells, and human hematopoietic cells, resulting in cytotoxicity, oxidative stress, immunomodulatory and inflammatory reactions, and further genotoxic potential, leading to a considerable increase in the levels of DNA damage and mutations.^45,178–180^ Understanding how nanoparticles interact with blood components is a crucial first step in evaluating their true hazard potential.

Micro/nanoplastics (MNPs), hemoglobin (Hb), hematopoietic stem cells (HSCs), red blood cells (RBC), white blood cells (WBC), endothelial cells (EC cells), reactive oxygen species (ROS), interleukin (IL), tumour necrosis factor (TNF), differentially expressed genes (DEG), C-reactive protein (CRP), alanine transaminase (ALT), alkaline phosphatase (ALP), aspartate transaminase (AST), lactate dehydrogenase (LDH), creatinine phosphokinase (CPK), colony forming units (CFU), cardiovascular (CV), oxygen (O_2_), nitric oxide (NO), glucose (glu), immunoglobins (Igs), phosphorous (P), calcium (Ca).

**Fig. 2:**
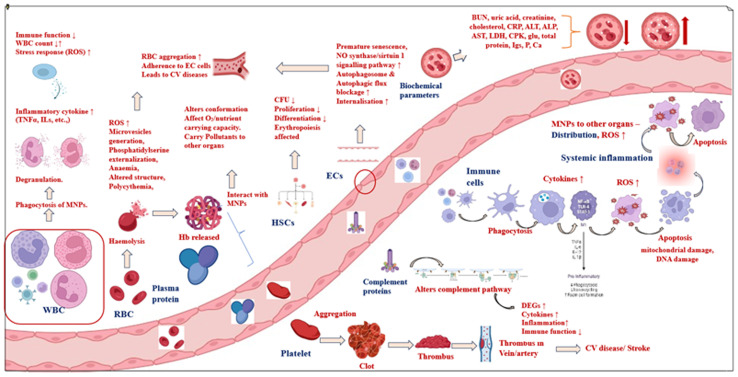
Interactions and negative effects of MNPs on blood components.

This paper aims to review the existing literature on the toxicity of micronanoplastics on blood components (Fig. 2).

### 7.1. Effect on plasma proteins

The liquid portion of blood, or plasma, is made up of several proteins, such as albumin, globulin, and fibrinogen. These proteins are essential for maintaining the osmotic balance of blood, carrying nutrients, and producing blood clots.^181^ Although our knowledge of the effects of MNP exposure on many human cells (such as cell viability, chromosomal damage, toxicity, *etc.*) is constantly growing, there is relatively little data on the hazard potential of MNPs on human proteins.^57^ Hence, it is high time to focus on studies of the impact of MNPs on proteins. Fundamental bio-macromolecules called proteins are essential for cellular and organismal processes. Upon entry into the physiological context, MNPs bind with proteins immediately. Ligand–protein binding is the key factor in defining the toxicokinetics of any hazardous substance. When a chemical is consumed by an organism, its toxicokinetics are described through the measurement of its ADME: absorption, distribution (in the broadest sense), metabolism, and excretion. Toxicokinetics is a branch of toxicology that studies the destiny of a chemical in the body to calculate the amount of the poisonous substance (either parent molecule or metabolite) that reaches the portion of the body where it will exhibit its toxic impact within a given timeframe.^182^ Toxicokinetics information aids in better estimating health risks and can also aid in lowering the number of tests required to determine the hazardous effects of a chemical. The risk assessment methods for harmful compounds, such as toxicity testing, dose selection, and hazard assessment by comparing experimental animal *versus* human systemic exposure, might benefit from a base set of necessary kinetic data.^183^ These chemicals are absorbed into the human body internally *via* ingestion, inhalation, and dermal exposure. After absorption, inhaled substances enter the lungs, from where they enter the bloodstream directly and travel to the heart, which is the next organ after the lungs. Ingested substances entering the gut also arrive in the bloodstream and reach the liver as the first organ.^182^

The second phase in toxicokinetics, *i.e.*, the distribution of a substance and its metabolites inside the body, is influenced by three major factors, namely,

(1) The affinity of the substance for plasma proteins,

(2) The partitioning between blood and specific tissues and,

(3) The capacity of the substance to pass through biological membranes (*e.g.*: the blood–brain barrier (BBB) and the blood–placental barrier (BPB)).^184^

The main element among these that affects the bioavailability and distribution of hazardous substances is plasma protein binding. A toxicokinetic investigation can rule out systemic toxicity if the plasma proteins do not bind to the MNPs. The systemic toxic effects can be anticipated to be minor if the binding is poor and the impacts on protein structure are extremely small; hence, toxicokinetics investigations can exclude systemic toxicity studies and suggest focus on other organ toxicity studies instead. However, strong protein binding prevents high-molecular-weight protein–ligand complexes from crossing biological membranes, resulting in impaired metabolism and renal clearance, which may be a sign of persistence and/or bioaccumulation.^49^ Interactions that are physical or chemical between these plastics and proteins (particularly plasma proteins) are the basis for the negative impacts of MNPs on human cells. MNP–protein complexes have an impact on the subsequent biological reaction. Hence, studies of the particular interactions between MNPs and various proteins must be investigated further in order to provide a foundational study that aids in the toxicokinetics and toxicodynamic profiles of these hazardous chemicals. Recent research has demonstrated that once MNPs enter the bloodstream, they form a protein coating known as a “protein corona”, which modifies the physicochemical characteristics of the MNPs, such as their size, surface charge, and hydrophobicity, and also determines their fate in the body, including circulation time, distribution, uptake and internalization in cells, and toxicity.^185,186^ The formation of “hard” or “soft” coronae is yet another topic under investigation in this regard. The term “soft corona” refers to the creation of layers of extremely complex biomolecules that interchange quickly, whereas “hard corona” refers to a long life and low complexity balance.^187,188^

There are well-accepted *in vitro* methods to measure protein binding.^189,190^ These methods include mass spectrometry (to pinpoint the precise plasma proteins and associated amino acid residues that interact with ligand);^191^ spectroscopic methods including UV-vis, fluorescence, and circular dichroism spectroscopy (to investigate the modifications in protein structure^192^ brought on by the interaction with the ligand); electron microscopy (to visualize how MNPs interact with plasma proteins);^193^ size-exclusion chromatography and high-performance liquid chromatography to separate proteins from ligands and analyze their binding.^194^ However, in the last two decades, fluorescence spectroscopy and its calculations to measure protein binding have revolutionized these research areas. Weak, moderate, or strong protein–ligand binding can be found from the Stern–Volmer constants, quenching constants, binding constants, and thermodynamic constants, which are calculated from the results of the fluorescence emission and excitation profiles of a particular binding protein.^195,196^

Fluorescence spectroscopy, resonance scattering spectroscopy, UV-visible (UV-vis) absorption spectroscopy, circular dichroism (CD), and Fourier transform infrared (FT-IR) spectroscopy were used to investigate the impact of polyvinyl chloride microplastics (PVC MPs) on bovine serum albumin (BSA) and human serum albumin (HSA) under simulated physiological conditions. PVC MPs alter the microenvironment and secondary structure (decrease in α-helix) of both proteins *via* electrostatic interaction.^197,198^ Interaction analysis of functionalized polystyrene nanoplastics (unfunctionalized: PS, carboxy: PS-COOH, and amine: PS-NH_2_) with human hemoglobin (Hb) applying multi-spectroscopic and docking methods revealed that all the NPs bind with the hydrophobic pocket of the β-chain in Hb; PS and PS-NH_2_ interact *via* hydrophobic forces, while PS-COOH binds *via* hydrogen bonding (predominantly) and van der Waals force, as validated from docking results. PS-NH_2_ binds effectively and changes the conformation of Hb by increasing the hydrophobicity around aromatic residues, particularly tryptophan.^199^ A similar investigation using human fibrinogen (HF) revealed that nanoplastics disturb the structure of HF in a concentration-dependent manner, with PS-NH_2_ having the greatest effect.^200^ Fluorescence correlation spectroscopy was used to assess the amount of transferrin adhered to sulfonate (PSOSO_3_H) and carboxyl- (PSCOOH) polystyrene NPs. The secondary layer (soft corona) exhibits dynamic exchange since it is reversibly bound, but the first layer (hard corona) is irreversibly bound. This suggests that an exposure memory effect may exist in the NP corona.^201^ This binding strategy at the molecular level should be explored for many more proteins so that a clear database of the distribution of NPs by proteins in the human body can be visualized and utilized for toxicokinetics studies.^202^

### 7.2. Effect on hematopoietic cells

Hematopoietic stem cells (HSCs) are multipotent progenitors (common ancestors) that can regenerate all of the various cell types that make up the blood-forming system, and are capable of self-renewal.^203^ Since they are the base cell of the entire immune and blood systems, they are extremely sensitive to hazardous substances.^204^ Both bone marrow and peripheral blood contain HSCs, which can be differentiated into all of the adult functional blood cells, in lines known as myeloid and lymphoid cells. Myeloid cells include neutrophils, macrophages, basophils, eosinophils, monocytes, erythrocytes, and megakaryocytes to platelets. Lymphoid cells include T cells, B cells, natural killer cells, and innate lymphoid cells. Dendritic cell development involves both lymphoid and myeloid lineages.^205^ Studies have verified that exposure to MPs and NPs can impair mammalian hematopoiesis. Three different types of human hematopoietic cells—Raji-B (as a model for B-lymphocytes), THP-1 (as a model for macrophages), and TK6 (as a model for lymphoblastoid cells)—have been used to examine the impact of polystyrene nanoplastics. Examination using TEM, confocal microscopy, and flow cytometry revealed that B-lymphocyte cells internalize PSNPs more than the other two models, and that the internalization is dependent on both particle size and cell type. Additionally, PSNPs promote cytotoxicity and cell stress by producing free radical ROS (reactive oxygen species) in blood cell lines. In Raji-B and THP-1 cells, PSNPs also caused the loss of mitochondrial membrane potential (MMP).^206^ Even at lower concentrations of 0.2 mg mL^−1^, 80 nm PSNPs cause haematotoxicity in human CD34+ HSCs *via* cell internalization, elevated ROS, and lactate dehydrogenase (LDH) production, which results in cell death. Additionally, exposure to PSNPs alters the concentrations of metabolites such as amino acids, short-chain fatty acids (SCFAs), organic acids, fatty acids, and carbohydrates, primarily impacting the citrate cycle (TCA cycle) metabolism pathway in HSPCs. PSNPs strongly upregulated eight metabolites (dimethylglycine, propionic acid, oxoglutaric acid, nonanoic acid, linoelaidic acid, 2,2-dimethyl adipic acid, and glucose 6-phosphate) and considerably downregulated two metabolites (ethylmethylacetic acid, isocaproic acid).^207^ Colony-forming assay demonstrated that 5 μm of PSNPs could impact peripheral blood cell count by preventing the differentiation of bone marrow HSCs into granulocytes and megakaryocytes. Transcriptome analysis of mouse BM cells using RNA-sequencing technology revealed that genes involved in T cell homeostasis, osmotic stress response, extracellular matrix, and structural organization were altered, and metabolic pathways such as Jak/Stat, fatty acids, and the pentose phosphate pathway NADP and nucleotide metabolism were all impacted.^174^ After differentiation and maturation in the thymus, T cells are distributed throughout the immunological organs *via* lymphatic and blood circulation to conduct immune activities. T cell homeostasis disruption will have an effect on the adaptive immune system, since it regulates lymphocyte development.^208^ Dysregulation of Jak/STAT signaling in humans will result in a variety of blood cell diseases and malignancies (leukemia).^209^ Multi-omics analysis using 16S rRNA, metabolomics, and cytokine chips to investigate potential hematopoietic system mechanisms in C57BL/6J mice demonstrated that intragastric administration of PSMPs and PSNPs with sizes of 10 nm, 5 nm, and 80 nm at a dose of 60 g for 42 days caused changes in gut microbiota, metabolites, and cytokines, which affected the proliferation and differentiation of HSCs.^210^

Thus, exposure to MNPs impaired the ability of HSCs to self-renew, proliferate, and differentiate indefinitely, which led to disorders of the hematopoietic system. These results are useful in assessing the toxicity pathways and metabolite indicators of hematopoietic cell damage due to nanoplastic exposure, which can set the groundwork for early toxicity assessment and prevention.

### 7.3. Effect on red blood cells

Erythrocytes, which often known as red blood cells (RBC), are the part of blood that actually carries nutrients and gases throughout the body. The adult erythrocyte is anucleate and has a biconcave discoid form. Their configuration enables them to move through the circulatory system and provide adequate gas exchange. The lifespan of a red blood cell is approximately 120 days. It must perform the necessary purpose in that brief period of time. RBCs are subjected to attacks throughout their lifespan, which cause continual morphological and physiological alterations.^51^ Changes in RBC morphology or a reduction in their lifespan will have health effects, and their assessment can reveal details about the general well-being of a person. Research points to oxidative stress and inflammation of erythrocytes in chronic inflammatory disease.^211^ Furthermore, the presence of nucleated red blood cells in circulation may be a sign of hemorrhage, hypoxia, hemolysis, and the presence of leukemia or other malignancies.^212^ The hemolytic potential of nanomaterials is well acknowledged,^211,213,214^ and since prolonged hemolysis can have deadly health consequences, it is crucial to evaluate the hemolytic potential of each nanomaterial. Research on the effects of MNPs on red blood cells is still in its early stages; thus, additional studies are required to properly understand their effects. However, various studies have implied that RBCs may be adversely affected by microplastics. Polystyrene microplastics can significantly enhance the generation of ROS, cause oxidative stress in RBCs and impact their overall functions. The RBCs of mice developed polycythemia in response to PSMPs, accompanied by a reduction in membrane elements, an alteration in bilayer thickness, and an increase in intrinsic lipid curvature. These findings indicated RBC membrane function impairment.^215^ In a different *in vivo* experiment employing the C57BL/6 mouse model, animals were given daily 6, 60, or 600 μg per day doses of polyethylene microplastics for 15 days. The outcomes showed that the typical structure of their RBCs was significantly altered by exposure to 600 μg per day of MPs, leading to a variety of aberrant forms.^216^ When Nile tilapia (*Oreochromis niloticus*) were exposed to MPs, they experienced a huge increase in eryptosis (apoptosis), poikilocytosis (shape aberrations), and nuclear anomalies of RBCs.^217^ Among various surface functionalized PSNPs (pristine, COOH, and NH_2_), amine-modified PSNPs with a size of 100 nm induced greater hemolysis with structural changes in human RBCs (since cell mediators, such as ATP, calcium, and GSH, were disturbed by the PSNPs), phosphatidylserine externalization, microvesicle generation and distress in the intracellular microenvironment. Similar results were observed in rat RBCs, showing no inter-species differences.^60^ Plastic additives can seep into the environment or food matrices and enter the human body. As an example, phthalates enter the body by ingestion, are absorbed into the bloodstream, and produce several abnormalities in erythrocytes, such as eryptosis, hemolysis, membrane aberrations, *etc.*^218^ In addition to the toxicity of pristine MNPs, the impact of co-pollutants should be considered. One study reported that cadmium and PSMPs induced disruption of the membrane components and lipid bilayer of RBCs.^219^ Several other investigations demonstrated the toxic impact of MNPs in reducing RBC cell count in various species.^220–222^

### 7.4. Effect on white blood cells

White blood cells (WBCs), which are often referred to as leukocytes, are generated in the bone marrow and enter the bloodstream to serve a critical protective function in defense against infections, foreign objects, and aberrant cells. White blood cells include monocytes (differentiate into macrophages and dendritic cells: carry out phagocytosis), lymphocytes (B and T cells: control immune response), neutrophils (first line of defense against infections), eosinophils (release enzymes that harm infected cells), and basophils (involved in defense mechanisms by presenting histamine and other chemicals and promoting inflammation). The health of a person can be inferred from the quantity and distribution of these cells. Medical problems might be indicated by an aberrant WBC count, either excessively high (leukocytosis) or excessively low (leukopenia).^53^ Studies have demonstrated that WBC count and function may be negatively impacted by exposure to micronanoplastics. The cell counts in five-week-old male C57BL/6 mice treated with 0.5 mg of 5 μm PSMPs were dramatically lowered according to toxicological tests.^174^ This reduced WBC count may be associated with the immunosuppressive effects of MNPs. In an *ex vivo* investigation, whole human blood samples were subjected to PSNP exposure, and it was discovered that PSNPs were internalized by monocytes and peripheral mononuclear cells (PMN), causing DNA damage and modifications in the whole blood secretome. The expression of several cytokines related to inflammatory, immunological, and stress responses, as well as cell proliferation, supported these findings.^178^ MNPs can thus cause cellular cytotoxicity, oxidative stress, and white blood cell growth/reduction, which implies that MNPs can elicit an immunological response and potentially impair WBC function. In a study on human peripheral lymphocytes, 250 and 500 μg mL^−1^ concentrations of 10–45 μm red fluorescent PEMPs markedly increased nucleoplasmic bridge formation (NPB), micronucleation (MN), and nuclear bud development (NBUD), which indicated genomic instability in lymphocytes.^46^ WBCs such as neutrophils and macrophages, which are referred to as phagocytes, can engulf, form phagosomes around, and “digest” antigens. The information from the antigens is then transmitted to the surface of the scavenger cells, where it is recognized by the adaptive immune system.^223^ When fish consume MNPs in an aquatic setting, there is evidence of cytotoxicity *via* enhanced neutrophil primary granule degranulation.^224^ When transgenic zebrafish (*Danio rerio*) larvae Tg(lyz:DsRed2) were exposed to PS-MNPs and the co-pollutant cadmium, their neutrophil expression was altered, particularly in the case of nanoplastics +cd, which induced high immune toxicity by generating higher ROS levels in the larvae.^225^ An *in vivo* study using C57BL/6 mice showed that PSNPs induce infiltration of macrophages, eosinophils, neutrophils, and lymphocytes in bronchoalveolar lavage fluid with further induction of cytokine generation followed by lung inflammation.^226^ Using rat basophilic leukemia (RBL-2H3) cells, it was discovered that PSNPs were internalized by basophils through interactions with cell membranes, endocytosis by clathrin- and caveolae-mediated pathways, and macropinocytosis. The PSNPs then became distributed in the cytoplasm and entered lysosomes before being released from cells through lysosome-mediated pathways and passive penetration.^227^ Similarly, 15 days after the exposure of tilapia (*Oreochromis niloticus*) to MPs, a histological study of the kidney indicated loss of basophilic cytoplasm in a number of tubules, indicating cytotoxicity in basophils due to MPs.^228^ In conjunction with the plasticizer di(2-ethyl)hexyl phthalate (DEHP), MPs significantly elevated oxidative stress, increased the production of cytokines and neutrophil extracellular traps (Nets), and blocked the *Wnt*/*β*-catenin pathway, which resulted in inflammation and skin fibrosis. Hence, co-pollutants mainly exhibit synergistic toxic injury with MNPs.^135^ In conclusion, research indicates that exposure to MNPs may detrimentally impact WBCs, including a decline in WBC count and interference with regular immune response processes.

In response to an influx of MNPs, WBCs and other immune cells such as natural killer cells, dendritic cells, and many others work together to release cytokines and other inflammatory mediators that cause tissue damage, organ infiltration, inflammatory response, and ultimately systemic inflammation.^229^

### 7.5. Systemic inflammation

Systemic inflammation is caused by the endocytosis or phagocytosis of infectious bacteria, non-infectious agents such as exotoxic pollutants, and auto-immune molecules by immune cells. These agents trigger the release of immune system chemicals such as prostaglandins, cytokines, *etc.*, leading to a cascade of reactions throughout the body, resulting in tissue damage and organ malfunction under chronic conditions.^230^ NLRP3 inflammasome is one such inflammatory signal activated by the phago/endocytosis of exogenous pollutants and rupture and release of lysosomal contents such as reactive oxygen species (ROS) and many other mediators. This inflammasome may be used as a sensor for detecting the immunotoxicity of MNPs.^231^ Polystyrene MNPs triggered ROS buildup and activation of the NLRP3 inflammasome in human primary macrophages,^232^ human monocytic (THP-1)^233^ and mouse lung (MLE-12) cell lines,^234^ and in mouse hepatocytes (AML12 cell line).^36^ Polystyrene MNPs have been proven to activate the NLRP3/caspase-1 signaling pathway in rat^235^ and mouse^236^ models *in vivo*, triggering inflammatory reactions and pyroptosis. The presence of co-contaminants (arsenic) with PSNPs induced the above pathway leading to pyroptosis in the mouse liver.^37^ NPs cause higher inflammation than MPs, as shown in one study in which NPs drove gut macrophage reprogramming and interleukin-1 (IL-1) production by inflicting lysosomal degradation. This IL-1 signaling from the intestine can influence brain immunity, resulting in Th17 differentiation and microglial activation, all of which are associated with a loss in cognitive function and short-term memory in mice.^40^ Female Sprague–Dawley rats that had inhaled polyamide MNPs showed elevated levels of proinflammatory cytokines such as IL-6, CRP, and MCP-1 (biomarkers of systemic inflammation) in their plasma 24 hours after the inhalation.^237^ More research into the systemic inflammation generated by MNPs is needed to determine the time course of this exposure and the inflammatory cytokines. When earthworms were exposed to palladium-doped polystyrene nanoparticles, accumulation of the NPs and a decreased development rate (21.3–50.8%), decreased antioxidant enzyme activity, and an increase in ROS were observed. By internalizing these NPs (about 0.12 μg per cell), earthworm immune cells caused lysosomes to rupture, which then triggered autophagy and ultimately led to cell death.^238^ Using RAW264.7 cell macrophages, 4 μg mL^−1^ of 191.6 nm polyethylene terephthalate (PET), 1.85 nm PET, and low-density polyethylene (LDPE), which were produced as real-world plastics^239^ from food containers and bottles, were examined for cell internalization. Macrophages serve as the first line of defense against any foreign agents, and thus, the MNPs were taken up by the macrophages within 3 hours of incubation. The lysosomal activity was dramatically reduced by nano-PET particles; chronic modification of lysosomal activity and cellular homeostasis results in tissue degeneration and cellular damage.^159^ MALT1 expression was elevated in mouse lung tissue following co-exposure to HDM (house dust mites) and MNPs. Histamine-induced endothelial permeability and IgE-dependent mast cell cytokine production are mediated by MALT1, which is expressed in lymphoid, mast, and endothelial cells. This implies that co-exposure to allergens and MNPs may have a synergistically effect to intensify inflammatory conditions.^32^

### 7.6. Effect on platelets

Blood cells known as platelets or thrombocytes are produced from megakaryocytes and exist in circulation for 5–7 days. They play a role in stopping bleeding from injuries, which starts with platelet activation (shape change) and adhesion to the vessel wall below the endothelium, and the release of chemical signals that cause platelet aggregation. The coagulation cascade is subsequently triggered, resulting in the transformation of fibrinogen into fibrin, which creates a mesh-like structure to support the clot. The blood clot shrinks, bringing the frayed edges of the blood vessel together and closing the wound. Once the wound has healed, the clot must be broken up in order to allow normal blood flow. This procedure, known as fibrinolysis, entails the activation of enzymes that disassemble the fibrin meshwork.^54^ Proper platelet activity is critical for overall health. Abnormalities of the platelets cause either excessive clotting or excessive bleeding. Platelet-related disorders include hemophilia, von Willebrand disease, and thrombocytopenia (low platelet count).^55^ Researchers have reported the interaction of platelets with nanoparticles as they circulate in the blood, which induces a chain of chemical signals leading to fibrin and clot formation (thrombi) and the development of cardio- or cerebrovascular blockages.^240^ Hence, it is critical to understand the thrombogenic capacity of any external agents entering the body. In a previous study by Kim *et al.* in 2022, in addition to affecting RBCs, PSNPs also had an impact on platelets in humans, modulating the coagulation cascade, RBC adhesion, and thrombus generation. Administration of PSNPs in rats also proved to induce thrombus formation.^60^ First, 100 nm PSNPs and PS-NH_2_ were preincubated with either thrombin or fibrinogen in an *ex vivo* human thrombin/fibrinogen clot model, and fibrin clot formation was assessed using turbidity and thromboelastography (TEG). Microplastics have an inhibiting influence on the production of fibrin clots. When first incubated with thrombin, PS-NH_2_ significantly impacted clot strength and the rate of fibrin production; however, this impact was significantly lessened when they were preincubated with fibrinogen.^241^ In another study using a microfluidic-based *in vitro* thrombosis model, optical irradiation confirmed the tissue-injury-induced thrombosis following MNP exposure.^242^ When zebrafish were exposed to copper co-pollutants combined with PSMPs, they increased the expression of genes encoding platelets (f5, ahsg2, serpina1, tor4aa, aldoaa, igf2b, hgfa, serping1, tfa, *etc.*), which might activate large numbers of platelets, and caused aggregation, coagulation, and clot formation.^243^ In a real-time analysis, thrombi were collected from patients who underwent cardiac surgery and analyzed using Raman spectroscopy. Surprisingly, the spectral data revealed the presence of exogenous microplastics in human thrombi, which confirms the clot formation ability of MNPs in the bloodstream.^244^

### 7.7. Effect on vascular endothelial cells

Blood cells and proteins are frequently the main subjects of research in nanomaterial–blood interactions. However, it is also important to take into account the vascular endothelium in which these components are located. The endothelium is a thin layer of vascular endothelial cells that lines the inside of blood vessels. These cells are essential for maintaining blood vessel health and perform a variety of vital cardiovascular functions, including transporting nutrients and wastes across walls, regulating blood pressure, managing clotting and anticoagulation, controlling inflammation, and secreting hormones and enzymes for vessel function/coagulation/inflammation/maintaining vascular homeostasis, assisting in angiogenesis, and performing vasodilation and vasoconstriction using nitric oxide and endothelin.^245^ Vascular endothelial dysfunction, which is caused by high blood pressure or inflammation, contributes to a prothrombotic state and cardiovascular illnesses such as atherosclerosis/hypertension.^246^ Endothelial function is critical for overall cardiovascular health, and researchers continue to strive to comprehend the intricate relationships between endothelial cells and MNPs and their impact on health. The toxicological effects of inhaling polyamide MNPs (5 ± 1 μm) were evaluated in female 8–10 week-old Sprague–Dawley rats. The uterine vascular endothelial cells were affected, resulting in impaired vasodilation in microcirculation and elevated blood pressure.^237^ Among various functionalized polystyrene NPs (pristine, NH_2,_ and COOH), PS-NH_2_ and PS-COOH exhibited higher cytotoxicity toward the vascular endothelial cells and inflammatory response through the JAK1/STAT3/TF signaling pathway in mice, leading to coagulation dysfunctions and a prethrombotic state.^247^*In vitro* studies conducted on human cerebral microvascular endothelial cells (hCMEC/D3) showed that PS-NPs could enter the cells and trigger ROS generation, activate nuclear factor kappa-B (NF-κB), promote the synthesis of tumor necrosis factor (TNF-α), and cause necroptosis. This leads to a disruption of the tight junction of vascular cells, as seen from the decrease in transendothelial electrical resistance and reduced expression of occludin, which allowed for BBB passage and accumulation in the brain.^39^ In a recent study, researchers identified MNPs in fecal samples of patients with vascular calcification (VC).^248^ This is one of the pathological conditions in cardiac patients with the deposition of minerals in the vascular system, especially in the heart valves.^249^ Yan *et al.* reported in 2023 that PP-MPs and PSMPs were found in the feces of VC patients, and these particles induced mild VC in normal rats and intensified VC in vitamin D3 + nicotine-treated rats. Thus, MNPs in the vascular system can aggravate other pathological diseases in humans.^248^

### 7.8. Effect on complement proteins

The complement system is a collection of proteins that circulate throughout the blood and aid the ability immune system to fight illness by recognizing pathogens for phagocyte eradication. The native components are numbered C1, C4, C2, C3, C5, C6, C7, C8, and C9. C3 convertases are produced by complement activation, which cleaves C3 into C3b and C3a. Pathogens are marked for phagocytosis by C3b, which functions as an opsonin. Local inflammatory reactions are triggered by C3a, C4a, and C5a, which draw immune cells to the infection site. A membrane–attack complex made up of the terminal complement proteins assembles to create membrane holes. Increased vulnerability to infection or severe tissue damage results from deficiencies in the complement system or abnormal, unchecked complement activation.^250^ In freshwater benthic clams (*Corbicula fluminea*), PSMPs were reported to trigger the innate immune response by activating the complement and coagulation cascade pathways. In response to MPs, differentially expressed genes were noticeably enriched in these pathways. The major genes of the complement cascade system (*TIRINITY _ DN4384_ c0_ g1*, *TIRINITY _ DN4871_ c0 _ g1*, *TIRINITY _ DN1178 _ c0 _ g1*, *TIRINITY _ DN3052 _ c0 _ g4*, *TIRINITY _ DN14985 _ c0 _ g1*, and *TIRINITY _ DN20036 _ c0 _ g1*) were considerably elevated in the presence of MPs according to RT-qPCR results. Complement factor B (CFB), a part of the alternate pathway of the complement system, was also markedly increased. Additionally, the increased aggregation of pathogenic bacteria and the elicitation of hub genera demonstrated intestinal inflammation, supporting the MP activation of the complement and coagulation cascade pathways.^47^ In adult zebrafish (*Danio rerio*), exposure to PEMPs for seven days caused changes in the gut microbiome that increased the likelihood of infection in the intestinal mucosa. A dose-dependent increase in complement C3 and C4 content was observed when MPs activated the intestinal immune network pathway for the generation of mucosal immunoglobulins. The relative abundance of the *Plesiomonas* genus was favorably linked with the expression of immune-related genes (*pigr*, *il10*, and *ighv4-5*).^251^ Microplastic exposure can impair immune function by suppressing the apoptosis-related molecular pathways and calcium signaling of the complement system, among other immune-system-disrupting effects.^252^ These findings imply that microplastics affect the complement system and the capacity of the body to fight off foreign substances.

### 7.9. Effect on biochemical parameters

Biochemical parameters, which are chemical substances found in the blood, are indicators of health (including autoimmune illnesses, inflammatory conditions, and more) and organ health (such as the liver, kidneys, *etc.*). These include bilirubin, blood urea nitrogen, uric acid, creatinine, cholesterol, C-reactive protein (CRP), alanine transaminase (ALT), alkaline phosphatase (ALP), aspartate transaminase (AST), lactose dehydrogenase (LDH), and many others.^253^ Microplastics have been proven to alter hemato–biochemical parameters and produce anemia.^254^ An early juvenile Nile tilapia (*Oreochromis niloticus*) fish showed a significant increase in biochemical markers (creatinine, ALP, AST, ALT, glucose, uric acid, cholesterol, and total protein) after being exposed to microplastics for 15 days.^255^ Upon exposure to 500 and 1000 mg kg^−1^ MPs, the European pond turtle (*Emys orbicularis*) showed an increase in ALT, ALP, AST, creatinine phosphokinase (CPK), cholesterol, glucose, creatinine, urea, and calcium (Ca^2+^) activity and a decrease in LDH, triglyceride, total protein, albumin, total immunoglobulins, and phosphorus levels.^21^ Similarly, in common carp (*C. carpio*), following exposure to 0.4 mg L^−1^ of the herbicide paraquat and a combination of paraquat and microplastics, the AST, ALP, ALT, LDH, creatinine, CPK, glucose, and albumin levels increased, whereas the total protein, globulin, cholesterol, and triglyceride levels decreased, as well as the -glutamyl transferase activity. Based on these findings, higher concentrations of microplastics in the water considerably exacerbated the hazardous effects of the co-pollutant paraquat on fish.^256^ As a result, modifications in blood biochemical parameters may be a suitable biomarker to show the presence of tissue injury.

Among all the blood components discussed, plasma proteins, HSC, RBC, WBC, immune cells, platelets, and vascular endothelial cells are of particular interest. The impact on plasma proteins will lead to whole-body nutrient and oxygen deficiency. Further, alterations to the structure of HSA may impact its esterase enzymatic activity.^257^ This would impair the ability of HSA to bind to ester-containing hazardous compounds, which could lead to a decrease in the natural detoxification ability of the human body.^258^ WBCs and immune cells are the basis for the immunity of an individual. The destruction of these cells can pave the way for numerous immune deficiency diseases. Toxic impacts on RBCs, platelets, and ECs cells can lead to life-threatening cardiovascular and cerebral diseases. Hence, research should be conducted on these components to understand the extent of MNP toxicity and to take preventive measures. The severity of MNP toxicity can be evidenced by recent research in which microplastics (poly(methyl methacrylate)) were detected in the heart (left atrial appendage, epicardial adipose tissue, and pericardial adipose tissue) of cardiac surgery patients^259^ and MPs were detected in thrombi isolated from cardiac patients.^244^

## 8. Distribution to other organs and toxicity

Following their journey in the circulatory system, MNPs are dispersed throughout the organs, where they accumulate, disrupt metabolic pathways, elicit immunological responses, and, in more extreme cases, lead to immune dysfunction. Major organs like the lungs, liver, kidneys, and brain can be invaded by MNPs through blood circulation.^252^ Research on CD1 mice^260^ and C57BL/6 J mice^160^ has also proven that PS-MNPs were found to accumulate in the blood and other organs, *i.e.*, the spleen, lung, kidneys, liver, small intestine, heart, *etc.* Toxicity in the lungs was reported by Nemmar *et al.* in 2003 in hamsters; amine-PSNPs (60 nm) penetrated the pulmonary blood barrier, leading to pulmonary thrombosis and inflammation.^261^ A study conducted using PS-COOH with sizes of 40 and 200 nm to assess internalization by different organs indicated that the MNPs were taken up by HeLa (cervical cells), A549 (lung cells), and 1321N1 (brain cells) human cell lines.^262^ With a mean residence period of 17 days and a bioaccumulation factor (BCF) of ∼8, the gut had the highest level of exposure to 5 μm PSMPs.^263^ It was found that 0.1 μm PSNPs can enter hepatocytes from the bloodstream and cause DNA damage in the mitochondria and nucleus, which causes the dsDNA fragment to enter the cytoplasm and activate the DNA-detecting adaptor STING. The downstream cascade response was started by the activation of the cGAS/STING pathway. The NF-κB translocated into the nucleus and increased the release of pro-inflammatory cytokines, ultimately accelerating liver fibrosis.^35^ Studies on short-term absorption in mice using the oral administration of polystyrene MNPs (9.55 m, 1.14 m, 0.293 m) revealed that NPs enter the brain 2 hours after gavage. Protein molecules hindered the uptake of these NPs into the blood–brain barrier, whereas cholesterol molecules promoted it.^264^ MNPs are transferred to the placenta and filtered into the fetal organs, according to studies. Hesler *et al.* (2019) used BeWo b30 cells and 50 and 500 nm carboxy-modified polystyrene particles in their study and found weak embryotoxicity and non-genotoxicity at the concentrations tested (0.1–10 g mL^−1^), and no negative effects of NP exposure on placental barrier integrity.^265^ An *in vivo* study proved that carboxylate-modified polystyrene particles of 20–500 nm were dispersed in the fetal brain, lungs, and liver after crossing the mouse placenta.^42^ In 2020, using fluorescent optical imaging and hyperspectral darkfield microscopy, Fournier *et al.* reported the distribution of 20 nm rhodamine-labeled polystyrene beads in fetal liver, kidney, lung, heart, and brain tissues after intratracheal instillation of pregnant rats in late gestation. This finding suggests that NPs can migrate *via* the placenta and diminish fetal and placental weight, implying that exposure to plastic particles during pregnancy may have an impact on birth outcomes.^31^ In a recent fish study conducted in a realistic environment, MNP accumulation in the internal organs such as the gills (11.1 μm size of MNPs), heart (6.8 μm), kidneys (9.8 μm), and digestive system (25.9 μm) was observed, and the MNPs in the heart were in the same size range as hematocytes, which strengthens the crucial role of the circulatory system in the distribution of MNPs.^266^

## 9. Implications for human health

The toxic responses of various blood components to MNPs and co-pollutants (Table 2) were thoroughly addressed in the previous sections. It is critical to understand how these harmful responses will affect human health. Alterations in the conformations of plasma proteins due to MNPs affect their functions such as the transportation of nutrients, oxygen, minerals, drugs, *etc.*, which might lead to nutrient deficiency, oxygen deficiency, iron deficiency, drug inefficacy and many other effects. Cytotoxicity, genotoxicity, and the inability of HSC cells to differentiate and proliferate can result in impaired T-cell and B-cell hemostasis and hematopoietic disorders such as anemia, blood cancer, autoimmune diseases, *etc.* The hazardous effects on RBCs could result in hemolytic anemia, iron deficiency anemia, RBC aggregation, and aberrant clots in the body. Cytotoxicity, oxidative stress, cell organelle failure, apoptosis of WBC and immune cells, and the up- or down-regulation of cytokines and other inflammatory chemicals all contribute to immunological deficiency, inflammation in the blood and other organs, and systemic inflammation. Platelet abnormalities could result in thrombocytopenia (too few platelets in circulation), bruising, and persistent bleeding in the skin, gums, and other internal organs. The senescence or apoptosis of vascular endothelial cells along with RBC and platelet aggregation produces clots in blood vessels. These obstructions might cause cardiovascular disorders (*e.g.*, atherosclerosis) and brain ailments (*e.g.*, stroke). Alterations in the levels of biochemical parameters cause changes in overall body metabolism. Negative effects on complement proteins weaken immunity and increase the likelihood of infection. These implications to human health urge the necessity of further *in vivo* research and mitigation measures to control plastic pollution.

**Table 2:** Different MNPs and their toxic effects on various blood components

MNPs	Component	Model	Analysis done	Observations	Ref.
PENPS, PPNPs, PET-NPs, and nylon-6,6 (N66); each 5 nm	Tryptophan zipper		Molecular dynamics	MNPs change the secondary structure of the protein, prevent folding, and denature them	267
PSNPs	Human serum albumin	Human	Small angle neutron scattering (SANS)	Size of NPs plays a major role in the corona; larger particles form a soft corona due to decreased interaction	188
PSNPs-100 nm	Transferrin	Human	Size analyzer and fluorescence microscopy	PS-COOH – reversible binding, PS-OSO_3_H – irreversible binding. Adsorbed proteins encourage NP agglomeration	268
PSNPs-80 nm	HSA and immunoglobulin G	Human	Human *ex vivo* placental perfusion approach and shotgun proteomics	HSA binding facilitates PSNP transfer through the placenta	41
PSNPs – 100–500 nm	Lysozyme	Hen	Fluorescence spectroscopy	PSNPs bind lysozyme by hydrophobic interactions, promote amyloid fibril formation, and change the protein structure. Amyloid fibrils form at the interface and junction of the protein corona	269
PSNPs	Ubiquitin	Human	CD, NMR, and TEM analyses	PSNPs change ubiquitin structure, reduce ubiquitination by the hard protein corona	270
PSOSO_3_H and PS-COOH	Transferrin	Human	Fluorescence correlation spectroscopy	Secondary layer – soft corona – dynamic exchange – reversibly bound. First layer – hard corona – is irreversibly bound. Reveals exposure memory effect may exist in the corona	201
PSNPs, PS-COOH, and PS-NH_2_	Hemoglobin	Human	Multi-spectroscopic and docking methods	NPs bind with B-chain in Hb, PS and PS-NH_2_ – hydrophobic force PS-COOH – hydrogen bonding and van der Waals force. PS-NH_2_ influences the structure more. Disturbs functional structure, and might affect oxygen carrying capacity	199
PSNPs, PS-COOH, and PS-NH_2_	Fibrinogen	Human	Fluorescence spectroscopy	NPs modify conformation, with PS-NH_2_ having the highest effect	200
PVC-MPs	Serum albumin	Bovine and human	Multi-spectroscopic studies	MPs change the structure (decrease in α-helix) of both proteins by electrostatic interaction	197 and 198
PSNPs – 100 nm	Serum albumin	Human	Multispectroscopic and docking analysis	Alters secondary structure, affects esterase-like enzyme activity, and might affect nutrient transfer capacity	271
PSNPs (50, 200, and 500 nm)	TK6 – human lymphoblastoid cell line. THP-1 – human leukemia monocytic cell line. Raji-B – human B lymphocyte cell line	Human cell lines	Cell internalization and toxicity assays	Raji-B and THP-1 cells' uptake of PSNPs is greater. Loss of mitochondrial membrane potential – observed for Raji-B and THP-1 cells	206
PS-MNPs, 10 μm, 5 μm and 80 nm	HSCs	C57BL/6J mice	Colony-forming cell (CFC) assays	Reduced CF unit-granulocyte–macrophage (CFU-GM), and CFU granulocyte, erythrocyte, monocyte, macrophage (CFU-GEMM). MNPs impacted the proliferation and differentiation of HSCs and hindered erythropoiesis	210
PSNPs – 80 nm	CD34+ HSCs	Human	Cell internalization and toxicity assays	Increased ROS, LDH, cell death, alteration in the metabolite concentrations affecting the TCA cycle in HSCs	207
PSNPs – 5 μm	Bone marrow cells	C57BL/6 mice	CFC assay, transcriptome analysis – RNA-sequencing technology	Prevents bone marrow HSC differentiation. Genes involved in the extracellular matrix, T cell homeostasis, structural organization, and osmotic stress response were modified, and metabolic pathways such as fatty acids, Jak/Stat, pentose phosphate, NADP, and nucleotide were all affected	174
PSMPs and PSNPs – 10 nm, 5 nm, and 80 nm	Hematopoietic system mechanisms	C57BL/6J mice	Multi-omics analysis using 16S rRNA, metabolomics, and cytokine chips	Gut microbiome, metabolites, and cytokines were abnormal, which affected the multiplication and differentiation of HSCs	210
PS-NPs 50–250 nm	RBCs	Human	Hemolysis assay	PSNPs haemolyzed RBCs. The rate depends on the concentration and size of PSNPs	272
PSNPs, PS-COOH, and PS-NH_2_ – 50 nm	Venous blood	Human	Hemolysis assay	PS-NH_2_ damages the RBC membrane more than the other two	273
PSMPs – 1 μm	RBCs	Mice	Cell count and lipid profile	Enhanced ROS in RBCs, causes polycythemia, alters bilayer thickness, and increases intrinsic lipid curvature	215
Irregular PEMPs – 1–100 μm	RBCs	Sheep	Hemolysis assay	Rough structure causes greater hemolysis	274
PEMPs	RBCs	C57BL/6 mouse	Morphological analysis	RBC structure altered by PEMPs	216
PS, PS-COOH and PS-NH_2_	RBCs	Human and rat	Hematological analysis	PS-NH_2_ induced greater hemolysis, phosphatidylserine externalization, microvesicle production, and stresses in the intracellular microenvironment. Similar results were observed in rat RBCs	60
PEMPs	RBCs	Korean bullhead fish, *Pseudobagrus fulvidraco*	Hematological parameters	Decrease in RBC cell count	222
PVC-MPs	RBCs	Fish, *Etroplus suratensis*	Blood biomarker test	Decrease in RBC cell count	220
PSNPs 41 nm and PCNPs 158.7 nm	Neutrophils	Fathead minnow	Neutrophil function assays	Degranulation of neutrophils and neutrophil extracellular trap release. Increased ROS. Interfere with immune function	224
MNPs	WBCs	Human peripheral blood, mouse blood	Flow cytometry, fluorescence techniques, and nanocytometry	Damage phagocytic activity and affect immune function and health. MNP accumulation is influenced by age and environmental factors	275
PS-MNPLs, 10 μm, 5 μm and 80 nm	Lymphocytes	C57BL/6J mice	Blood cell count	Cell count upregulated. Implies inflammation or allergic reaction	210
PSNPs (50, 100, 310 nm), PVC_polyd_. PMMA_polyd_, PS_polyd_	Monocytes and dendritic cells	Human blood	Cytokine analysis	Alters IL-6, TNF, and IL-10 secretion. Provokes inflammation	276
PEMPs	Neutrophils and lymphocytes	C57BL/6 mouse	Hematological parameters	Neutrophil to lymphocyte ratio (N/L) increased—indicates inflammation	216
PSMPs – 5 μm	WBCs	C57BL/6 mice	Cell counting	WBC counts reduced	174
PSNPs	Whole blood	Human	Toxicological studies	MNPs are internalized by monocytes and peripheral mononuclear cells (PMN), causing cytotoxicity, genotoxicity, and cytokine release	48
Sulphate-modified nanoplastics (S-PSNPs)	Macrophages	Human and murine	Cell line studies	Lipids accumulated in cytoplasm and lysosomes impair lipid metabolism and differentiate macrophages into foam cells, which might lead to atherosclerosis	277
PSMPs – 100 nm	Inflammatory cytokines	Sprague–Dawley rats	Histological and gene sequencing analysis	IL-6, TNF-α and IL-1β upregulated. Modification in gene expression. Lung inflammation	278
PSNPs – 25 nm 70 nm	Cytokines	Human alveolar epithelial A549 cell line	Cell line studies and gene transcription and protein expression	IL-8, NF-κB, and TNF-α upregulated. Affects cell viability, apoptosis, and the cell cycle of lung cells	32
PS-MNPLs, 10 μm, 5 μm and 80 nm	Cytokines in plasma	C57BL/6J mice	High-throughput cytokine microarray	Cytokine alterations in plasma (IL-10, IL-12p40, IL-17A, eotaxin, MIP-1beta, IL-12p70) and inflammation	210
PSNPs – 50 nm	Immune response	Golden cuttlefish (*Sepia esculenta*)	Transcriptome data	16 key immune-related differentially expressed genes were altered, confirming the immune response for PSNPs	279
PSNPs – 50 nm, coexposed with Au ions	Embryos	Zebrafish	Embryo assays	Synergistic toxicity: mortality/underdeveloped hearts and yolk edema due to pro-inflammatory cytokines and mitochondrial damage	280
PSNPs, PS-NH_2_ – 100 nm	Thrombin/fibrinogen clot	Human *ex vivo* model	Turbidity and thromboelastography (TEG)	Inhibitory effects on fibrin clot formation with greater effect by PS-NH_2_	241
PSNPs, PS-COOH and PS-NH_2_ – 50, 100, and 500 nm	Clotting cascade	Human whole blood	Thromboelastography	PS-COOH activated clotting cascade, increased fibrin polymerization rates, and improved clot strength in a size- and concentration-dependent manner. PS-NH_2_ (100 nm particles) resembled PS-COOH except in 500 nm PS-NH_2_	58
PSNPs, PS-COOH, and PS-NH_2_ – 50 nm	Venous blood	Human	Platelet aggregation	PS-COOH caused platelet aggregation by upregulation of adhesion receptors. PS-NH_2_ caused platelet aggregation by perturbation of the platelet membrane	273
100 nm PSNPs	Umbilical vein endothelial cells	Human	Cell internalization and cytokine analysis	PSNPs taken up by EC cells, releasing autophagosome and blocking autophagic flux	281
100 nm PSNPs	Embryonic chorions	Zebrafish (*Danio rerio*)	Fluorescence assay	Causes local hypoxic microenvironment, affecting EC cells and thus development of embryos	282
PSNPs – 50–250 nm	RBC and EC cells	Human	Hemolysis and fluorescence assay	PSNPs promote hemolysis and RBC aggregation and enhance their adherence to EC cells, which may cause cardiovascular diseases	272 and 283
PSMPs −1 μm	*In vivo* – IV injection	C57BL/6N mice	Cytokine assay	Higher expression of cytokines IL-1β and Icam-1 in aortic vessels	284
PSNPs − 25 nm	Coronary artery EC cells	Porcine	Senescence-associated β-galactosidase (SA-β-Gal) assay	Premature senescence of EC cells by nitric oxide synthase/sirtuin 1 signaling pathway	285
PEMPs	Biochemical parameters	C57BL/6 mouse	Metabolic profiling	Elevated AST, ALT, serum glucose, creatinine, and total protein	216
PSNPs	Human blood cells and plasma proteins	Human	Multispectroscopic studies, gene toxicity, and cytotoxicity studies	Conformational changes in plasma protein and formation of protein corona. The coronated NPs caused higher genotoxic and cytotoxic effects in human blood cells (RBC, WBC)	286

## 10. Key findings

The potential consequences of the entry of micronanoplastics (MNPs) into the bloodstream *via* intravenous channels or barrier cells have been covered in this article. The following are key findings from the literature review.

(1) While circulating in the bloodstream, MNPs develop “hard” and “soft” coronae with plasma proteins (HSA, hemoglobin, transferrin, fibrinogen, *etc.*), which influence their structure and functionality. This will ultimately impact how nutrition, oxygen, medications, and many other substances are transferred throughout the body. For example, PSNPs alter the conformation of HSA^271^ and hemoglobin.^199^ In the area of toxicokinetics studies, more investigation into MNP–protein interactions is necessary.

(2) HSCs (hematopoietic stem cells) are essential for the regeneration of all blood cells. MNPs cause HSC cytotoxicity in addition to suppressing HSC proliferation and differentiation, which might result in hematopoietic diseases. Haematotoxicity in human CD34+ HSCs resulted from cell internalization, elevated ROS, and lactate dehydrogenase (LDH) production by MNPs.^207^ Furthermore, modification of the metabolic pathway of HSCs was validated by transcriptome and multi-omics analyses.

(3) In RBCs, MNPs cause hemolysis as well as additional risks including oxidative stress, structural changes, and functional impairments, which can result in hemorrhage, hypoxia, malignancies (polycythemia), and many other conditions. PENPs were found to alter the RBC structure in a C57BL/6 mouse model.^216^

(4) MNPs have the potential to negatively impact WBCs (monocytes, lymphocytes, neutrophils, eosinophils, and basophils), which could compromise the effectiveness of the immune system and raise concerns about general health. These negative impacts include DNA damage, alterations in cytokine expression, an increase in oxidative stress, changes in immunological response, disruptions in antigen processing function, and degranulation leading to inflammation and stress response. WBC count was drastically reduced in C57BL/6 mice treated with 0.5 mg of 5 μm PSMPs, which supports the above-mentioned effects.^174^

(5) MNPs have been discovered to be phagocytosed by immune cells, such as macrophages, as evidenced by an increase in the generation of proinflammatory cytokines (interferons, interleukins, tumor necrosis factors, NLRP3 inflammasome,^231^*etc.*) and prostaglandins. As a result, signaling pathways change, oxidative stress increases, and lysosomes rupture, which further leads to autophagy, cytotoxicity, pyroptosis, and organ inflammation, all of which can eventually result in systemic inflammation, tissue damage, and organ malfunction. More research is needed, however, to determine the overall degree of MNP-induced inflammation in the body.

(6) The effects of MNPs on platelets have been demonstrated to affect the coagulation cascade as well as events including red blood cell adhesion and thrombus (blood clot) formation. Platelets are necessary for preventing excessive bleeding/clotting and preserving overall health, but abnormal activity can result in coagulation disorders such as abnormal clotting or bleeding. For instance, carboxy PSNPs caused platelet aggregation *via* upregulation of adhesion receptors.^273^

(7) MNPs have been reported to decrease complement system pathways by modifying their genes and protein expression (for example, complement protein B increased in benthic clams due to PSNPs),^47^ hence compromising their involvement in immune function.

(8) The vascular endothelium, a crucial component of blood vessels, is essential for sustaining cardiovascular health and cerebral function. According to research, MNPs can impair vascular endothelial function by producing impaired vasodilation, elevated blood pressure, inflammatory reactions, and coagulation issues, which can contribute to cardiovascular disorders such as atherosclerosis and hypertension. For example, PSNPs affect tight junctions of human cerebral microvascular endothelial cells, possibly damaging the BBB.^39^

(9) MNPs alter the levels of hemato-biochemical parameters such as bilirubin, blood urea nitrogen, uric acid, creatinine, LDH, triglycerides, albumin cholesterol, C-reactive protein (CRP), and liver enzymes (ALT, ALP, AST), which may lead to a multitude of health problems, including autoimmune diseases and inflammation. Fish models presented significant increases in biochemical markers (creatinine, ALP, AST, ALT, glucose, uric acid, cholesterol, and total protein) when exposed to MNPs for 15 days.^228^

Briefly, this article has emphasized the possible toxicity of MNPs on blood components as well as the necessity for additional research in this field.

## 11. Research gaps in toxicological information and future perspectives

Although substantial progress has been made in the field of the toxicity of microplastics in blood, there are still many research gaps and areas that need to be further explored. They are as follows:

(1) Realistic exposure: The most significant research gap is the lack of toxicity data for real-world MNPs (true-to-life plastics). More research is required to mimic real-world exposure settings, including examining the root cause of MNP contamination in the bloodstream and any potential health effects.

(2) Combined toxicity of MNPs and co-pollutants: Despite scientific evidence showing the deleterious impact of MNPs on the circulatory system, studies on the combined impacts of MNPs and co-pollutants are extremely rare. To determine how these co-contaminants and microplastics might interact and whether their combined presence worsens toxicity, more research is required.

(3) Toxicity of monomers: Nanoplastics can be degraded further to monomers. Polystyrene, for example, is made up of styrene monomers that are bonded together. Since PS is widely used in food containers, PSNPs and styrene monomers may leach into food and enter the human body. It has been discovered that this monomer is more harmful to mucous membranes, the respiratory system, the neurological system, and the reproductive system. Furthermore, the WHO has designated styrene as a potential human carcinogen.^287^ Similarly, the hazardous effects of various plastic monomers on ecosystems and humans, particularly blood components, must be investigated.

(4) Advanced technologies to determine toxicity: Methodologies such as *in silico* analysis, machine-learning algorithms, and ADMET analysis will aid in the detailed assessment of the binding of MNPs and their toxicological profile in the human body. ADMET studies include experiments to determine the adsorption, distribution, metabolism, excretion, and toxicity of substances. For example, absorption through the blood-barrier barrier or GI tract can be predicted through the BOILED-Egg method.^288^

(5) Human studies: To date, *in vivo* toxicity studies have been conducted using animal models, and the results have been extrapolated to human health implications. Consequently, toxicity studies utilizing human-relevant experimental models must be conducted.

(6) Bioaccumulation: MNPs can enter the bloodstream and be transported to many organs and tissues according to studies. However, it is necessary to examine how they accumulate in the blood and other organs. In this context, in addition to acute exposure studies, a research gap exists regarding the potential chronic effects of MNPs in circulation. This research gap must be filled in order to calculate the bioaccumulation factor.

(7) Inflammatory pathways: Researchers are trying determine the pathways involved in the toxic effects of MNPs, for example, the JAK1/STAT3/TF and Wnt/β-catenin pathways. However, numerous pathways have yet to be identified, and further exploration is required.

(8) Immunological response: Regarding immune system toxicity, a basic understanding of how MNPs alter immunological responses, enhance oxidative stress, trigger inflammation, and decrease immune cell function has been established, but further research is necessary to fully understand how these effects relate to clinical disorders.

(9) Dose–response relationships: It is critical to establish dose–response relationships to ascertain the concentrations of MNPs in the bloodstream that may have a negative impact on health. To determine their influence, tests must be run with different MNP concentrations.

(10) Physiochemical characteristics of MNPs: MNPs with different characteristics, such as type, size, shape, and surface qualities, may have variable levels of toxicity. This aspect must be investigated in the bloodstream.

(11) Difference in toxicity among people: Further, the impact of microplastics in the bloodstream should be studied to see whether any particular demographic is more susceptible, such as young people, the elderly, or people with prior medical issues.

It is imperative to address these research gaps to achieve a complete understanding of the toxicity of MNPs and their complexes in the bloodstream and their potential impact on human health. This will also provide direction for the establishment of efficient risk assessment and mitigation measures.

## 12. Conclusion and outlook

MNPs have become a major environmental concern, raising concerns about their possible influence on the health of humans and other organisms. The hazardous effects of MNPs and their complexes with other pollutants on the aquatic and terrestrial environments have been proven in numerous studies. The negative impact of these particles on mammalian models and cell lines has been explicitly studied and the results are often expanded to human health. The current paper critically reviews technical investigations on the impact of MNPs and their co-pollutant complexes on blood and blood components. The presence of MNPs in a dynamic environment such as the bloodstream may have potentially deleterious effects due to interactions with diverse blood components, which represents a new area of research with substantial implications for human health. They have the ability to adsorb plasma proteins and complement proteins, as well as to form protein coronae, which can impact their action in biological systems. MNPs have been shown *in vitro* to have cytotoxic, genotoxic, and oxidative stress effects on blood cells. The reported scientific investigations have revealed the harmful effects of MNPs related to blood disorders such as anemia, thrombosis, cardiac diseases, and nutrient and oxygen deficiency, as well as immune system disorders. However, there are still many grey areas regarding the effects of MNP exposure in the bloodstream, due to the lack of studies on the combined toxicity of MNPs and co-pollutants, the effect of real-world MNPs, the effect of monomers and toxicological studies in humans, advanced methods to determine toxicity, bioaccumulation, inflammation, immunological pathways, and the impact of different concentrations of toxicants and their physiochemical characteristics. In this regard, this article has aimed to shed more light on the research gaps to be addressed immediately to better understand the extent and implications of the interaction between MNPs and blood components, as well as to create effective measures to lessen their potential negative impacts on human and environmental health.

## Abbreviations

MNPs: Micro/nanoplastics
MPs: Microplastics
NPs: Nanoplastics
PP: Polypropylene
PE: Polyethylene
LDPE: Low-density PE
HDPE: High-density PE
PVC: Polyvinyl chloride
PS: Polystyrene
PSNPs: Polystyrene nanoplastics
PSMPs: Polystyrene microplastics
PSMNPs: Polystyrene micronanoplastics
PEMPs: Polyethylene microplastics
PS-COOH: Carboxylated polystyrene nanoplastics
PS-NH_2_: Aminated polystyrene nanoplastics
PSOSO_3_H: Sulfonated polystyrene nanoplastics
PET: Polyethylene terephthalate
POPs: Persistent organic pollutants
PCBs: Polychlorinated biphenyls
BSA: Bovine serum albumin
HSA: Human serum albumin
Hb: Hemoglobin
HF: Human fibrinogen
HSCs: Hematopoietic stem cells
RBC: Red blood cells
WBCs: White blood cells
PMN: Peripheral mononuclear cells
EC cells: Endothelial cells
ROS: Reactive oxygen species
IL: Interleukin
TNF: Tumour necrosis factor
DEG: Differentially expressed genes
CRP: C-reactive protein
ALT: Alanine transaminase
ALP: Alkaline phosphatase
AST: Aspartate transaminase
LDH: Lactate dehydrogenase
CPK: Creatinine phosphokinase
TEG: Thromboelastography
UV: Ultraviolet
CD: Circular dichroism spectroscopy
NMR: Nuclear magnetic resonance imaging
TEM: Transmission electron microscopy

## Author contributions

Durgalakshmi Rajendran: investigation, data curation, writing – original draft, writing – review & editing. Natarajan Chandrasekaran*: conceptualization, supervision, resources, funding acquisition, writing – review & editing.

## Conflicts of interest

The authors declare that there is no conflict of interest.

## Supplementary Material
